# Strict Selection Alone of Patients Undergoing Liver Transplantation for Hilar Cholangiocarcinoma Is Associated with Improved Survival

**DOI:** 10.1371/journal.pone.0156127

**Published:** 2016-06-08

**Authors:** Hendrik T. J. Mantel, Andrie C. Westerkamp, René Adam, William F. Bennet, Daniel Seehofer, Utz Settmacher, Francisco Sánchez-Bueno, Joan Fabregat Prous, Emmanuel Boleslawski, Styrbjörn Friman, Robert J. Porte, European Liver and Intestine Transplant Association (ELITA)

**Affiliations:** 1 Department of Hepato-Pancreatico-Biliary Surgery and Liver Transplantation, University of Groningen, University Medical Center Groningen, Groningen, the Netherlands; 2 Department of Hepatobiliary Surgery, Hôpital Paul Brousse, University of Paris-Sud, Villejuif, France; 3 Transplant Institute, Sahlgrenska University Hospital and Sahlgrenska Academy, University of Gothenburg, Gothenburg, Sweden; 4 Department of General, Visceral and Transplantation Surgery, Charité Campus Virchow, Berlin, Germany; 5 Department of General, Visceral and Vascular Surgery, University of Jena, Jena, Germany; 6 Department of Surgery and Liver and Pancreas Transplantation, Virgen de la Arrixaca Clinic and University Hospital, Murcia, Spain; 7 Department of Hepato-Pancreatico- Biliary Surgery and Liver Transplantation, University Hospital de Bellvitge, University of Barcelona, Barcelona, Spain; 8 Department of Digestive Surgery and Transplantation, Lille University Medical Center, University of Lille Nord de France, Lille, France; University of North Carolina School of Medicine, UNITED STATES

## Abstract

Liver transplantation for hilar cholangiocarcinoma (hCCA) has regained attention since the Mayo Clinic reported their favorable results with the use of a neo-adjuvant chemoradiation protocol. However, debate remains whether the success of the protocol should be attributed to the neo-adjuvant therapy or to the strict selection criteria that are being applied. The aim of this study was to investigate the value of patient selection alone on the outcome of liver transplantation for hCCA. In this retrospective study, patients that were transplanted for hCCA between1990 and 2010 in Europe were identified using the European Liver Transplant Registry (ELTR). Twenty-one centers reported 173 patients (69%) of a total of 249 patients in the ELTR. Twenty-six patients were wrongly coded, resulting in a study group of 147 patients. We identified 28 patients (19%) who met the strict selection criteria of the Mayo Clinic protocol, but had not undergone neo-adjuvant chemoradiation therapy. Five–year survival in this subgroup was 59%, which is comparable to patients with pretreatment pathological confirmed hCCA that were transplanted after completion of the chemoradiation protocol at the Mayo Clinic. In conclusion, although the results should be cautiously interpreted, this study suggests that with strict selection alone, improved survival after transplantation can be achieved, approaching the Mayo Clinic experience.

## Introduction

Hilar cholangiocarcinoma (hCCA) is a devastating cancer originating from the biliary epithelium at the confluence of the right and left hepatic duct. Radical surgical resection of the tumor is the only curative option with a chance for long term survival. Five year survival rates after tumor resection vary between 25–40% [1–6]and occasionally, five years survival rates above 50% have been reported in a subgroup of patients undergoing elaborate surgery consisting of extended hemihepatecomy with vascular resection.[7]

For patients with unresectable hCCA or hCCA arising in the setting of a chronic liver disease, liver transplantation theoretically enables maximum resection margins and cures an underlying parenchymal liver disease. Unfortunately, the early experience with liver transplantation for hCCA was disappointing due to low survival rates and because of shortage of donor organs it was generally acknowledged that hCCA was not an indication for liver transplantation.[8,9]

In the last decade, however, the issue has been reconsidered mainly because of the results of the Mayo Clinic group. The Mayo Clinic has developed a neo-adjuvant protocol consisting of multimodal chemoradiation therapy. Patients undergo a consecutive regimen of external beam radiation therapy together with intravenous fluorouracil (5-FU), followed by intraluminal brachytherapy and finally oral Capecitabine while awaiting liver transplantation.[10] The early reports were remarkably optimistic with 5 year survival rates above 80%.[10,11] In more recent publications the survival rates have been adjusted to 65–70%, but remain unprecedented.[12]

From the beginning, the protocol has also been subjected to criticism because two separate interventions are combined in one protocol: (a) strict selection of patients with early stage disease and (b) neoadjuvant chemoradiotherapy.[13–15] The question has emerged in the literature whether the results of the Mayo Clinic should be contributed to the selection procedure, to the neoadjuvant chemoradiotherapy, or to the combination of both.

The aim of this study was to investigate the value of strict patient selection alone on the outcome of liver transplantation for hCCA. For this goal, we have retrospectively applied the Mayo Clinic selection criteria (Table 1) on patients that have undergone liver transplantation for hCCA in Europe. The European Liver Transplant Registry was used to identify patients transplanted for hCCA.

**Table 1:** Mayo clinic criteria for inclusion in the transplantation protocol for hilar cholangiocarcinoma[15–17].

**Diagnosis**	Pathologically confirmed hilar cholangiocarcinoma *or* CA19-9 >100 ng/ml in the presence of a radiographically malignant stricture
**Tumor**	Tumor size < 3 cm
**Distant metastases**	Absence of distant metastases on CT (and/or MRI) and isotope bone scan
**Lymph node metastases**	Negative EUS-FNA of regional lymph nodes *and* negative staging laparotomy/hand-assisted-laparoscopy with biopsy of regional lymph nodes

Abbreviations: CA 19–9; carbohydrate antigen 19–9, EUS-FNA; Endoscopic ultrasonography-fine needle aspiration.

## Materials and Methods

### Patients

To identify patients who underwent liver transplantation for hilar cholangiocarcinoma, we used the European Liver Transplant Registry (ELTR), a regularly audited registry of patients who underwent liver transplantation in one of the 153 contributing European centers. A list was extracted from the ELTR database containing all patients that were transplanted between 1990 and 2010 for hCCA. There were 249 patients from 57 European centers. Twenty-seven centers transplanted ≤ 2 patients. The list provided only basic variables, insufficient for in-depth analyses. Therefore, all centers were contacted with a request to participate in the study. Centers were preferably addressed in their own language (English, Italian, French, Swedish, Dutch). Each center was asked to upload additional information regarding patient and tumor characteristics and transplantation outcome by completing a webbased electronic questionnaire (SurveyMonkey, Palo Alto, California, USA) (S1 Fig). The primary outcome of this study was overall patient survival, defined as the period between transplantation and date of death or last follow-up (July 15^th^, 2013).

This study was approved by and performed under the auspices of the Board of the European Liver and Intestine Transplant Association (ELITA), the governing society of the ELTR (S2 Fig). All patient data were retrospectively and anonymously analyzed and therefore informed consent was not necessary. This type of research is compliant with Dutch legislation and was retrospectively approved by our institutional Medical Ethics Review Board (S3 Fig).

### Statistics

Statistical analyses were carried out using IBM SPSS Statistics, (IBM, Armonk, New York, USA). The results are expressed as the means ±SD. Comparison of means was performed with the Student t-test for independent samples. Comparison of categorical variables was performed with the Chi-Square test and Fisher’s exact probability test. Five-year survival rates were calculated using the Kaplan-Meier method and the differences between groups were calculated using the log rank test. Univariate analyses were conducted for patient survival by Kaplan-Meier estimates of survival probabilities and the log-rank test for comparisons. A Cox proportional hazard regression model was used to analyze associations with patient survival in multivariable analysis. P values were two-sided and values of less than 0.05 were considered statistically significant.

## Results and Discussion

Twenty-one centers uploaded data of 173 patients in the electronic database, resulting in a response rate of 69%. All patients were transplanted between 1990 and 2010. Twenty-six patients were excluded from the database, 12 because they were erroneously coded in the ELTR (the indication for transplantation was not hilar cholangiocarcinoma) and 14 because hCCA was incidentally found after liver transplantation. A study group of 147 patients remained.

Eighty-two patients were transplanted in the first decade between 1990 and 2000 and 65 patients were transplanted between 2000 and 2010.

The status of the distal bile duct margin was established in 137 patients and was tumor free (R0 resection) in 125 patients (91.2%). Mean follow-up was 4.1 years (± 5.0).

### Mayo Clinic selection criteria

**Fig 1:**
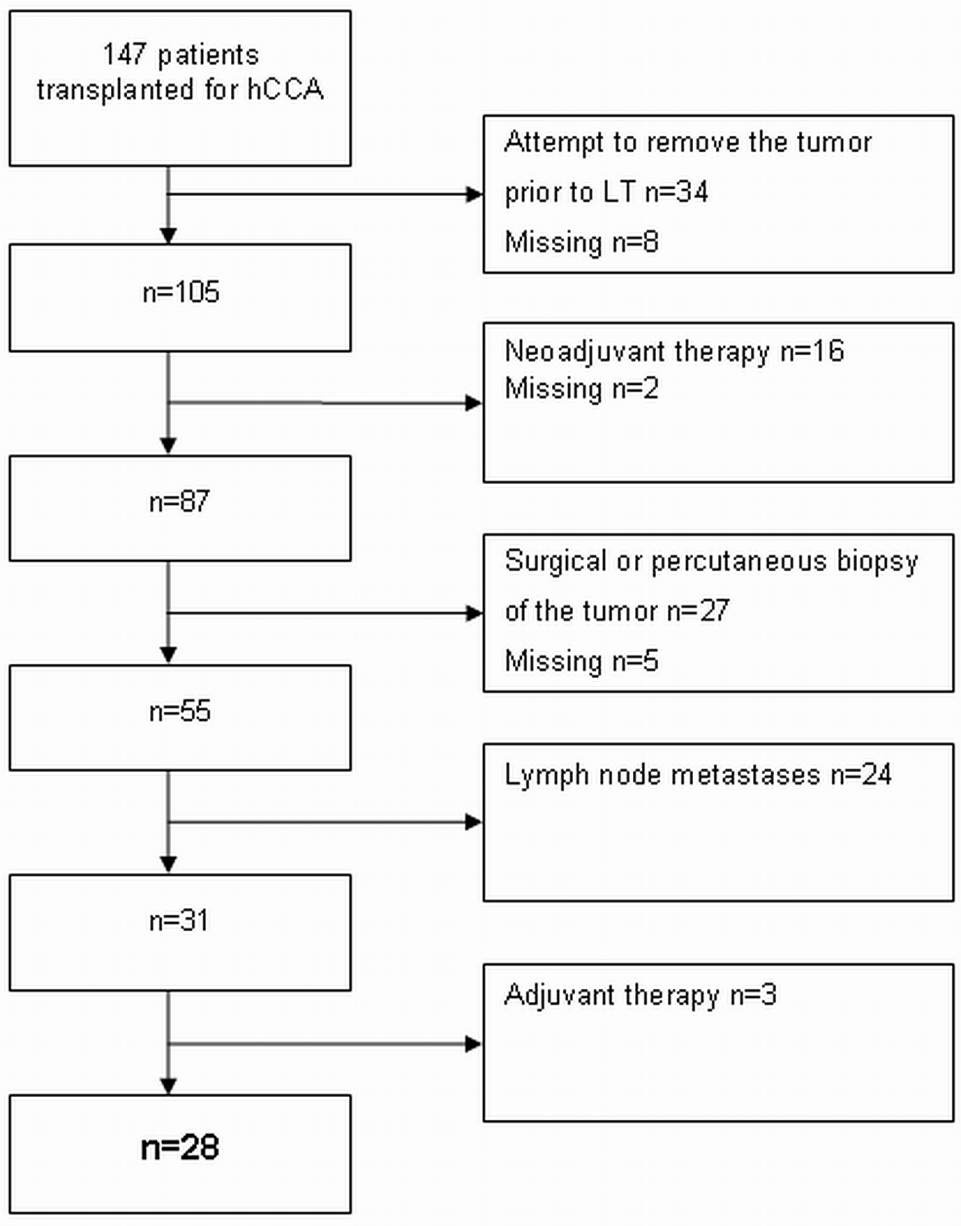
Flow chart illustrating the consecutive steps that were followed to select patients who met the Mayo Clinic criteria for liver transplantation, but were not treated with neo-adjuvant chemoradiotherapy.

The Mayo Clinic criteria for enrollment in the Mayo protocol were applied on our entire cohort of 147 patients (Fig 1). Patients with (an attempted) resection of the tumor prior to transplantation or percutaneous/surgical biopsy of the tumor were excluded. Endoscopic (brush) cytology was not an exclusion criterion. Patients with lymph node metastases were also excluded. The Mayo Clinic excludes patients with tumors >3 cm, but this does not correspond to a particular T-stage since T-staging is based on tumor infiltration depth rather than tumor size. Therefore, T-stage was not a part of the selection criteria in this study.

Because the aim of this study was to assess the outcome of patients who were not treated with neo-adjuvant chemoradiation therapy, 16 patients who did receive neo-adjuvant treatment were excluded. Neo-adjuvant therapy consisted in 3 cases of monotherapy (brachytherapy in 2 cases and external beam radiotherapy in 1 case) and in 10 cases of combination therapy: gemcitabine/oxaliplatin with radiotherapy (n = 4), Capecitabine and radiotherapy (n = 3), Mayo protocol (n = 2) and not specified (n = 1). In three cases the type of neo-adjuvant therapy was not specified.

Ultimately, 28 patients (19%) who had not been treated with neo-adjuvant chemoradiotherapy, complied with the Mayo Clinic criteria for liver transplantation. The clinicopathological variables of the group complying (group A) and not complying with the Mayo Clinic selection criteria (group B) are summarized in Table 2. There were no differences between the groups, except for the variables on which the selection was based. Adjuvant therapy was administered in 12 patients in group B, consisting of chemotherapy in six patients (3 patients with 5-FU, one with Gemcitabine/oxaliplatin, one with Mytomicin and one not specified), radiotherapy in one patients and a combination of chemotherapy and radiotherapy in five patients (not specified; n = 5). We determined the use of mTOR inhibitors in postoperative immunosuppressive regimens because of their potential anticancer effect. mTOR inhibitors were used in 11% of cases in group A versus 13% in group B (P = 0.77). Data on the presence of PSC was available for 25 patients in group A: six patients (24%) had underlying PSC.

**Table 2:** Clinicopathological variables of patients undergoing liver transplantation for hilar cholangiocarcinoma. Thirty four patients who underwent an attempt to surgically remove the tumor prior to transplantation and 8 patients with missing variables were excluded.

Variable	Patients transplanted for hilar cholangiocarcinoma n = 105		P-value
	Group A n = 28 Patients complying with the Mayo Clinic selection criteria for LT, without neo-adjuvant therapy	Group B n = 77 Patients not complying with the Mayo Clinic selection criteria.	
**Mean age in years (± SD)**	46 (± 9)	51 (± 10)	0.62
**Gender:**			
Male	18 (64%)	55 (71%)	0.48
Female	10 (36%)	22(39%)	
**Neo-adjuvant therapy**			
Yes	0 (0%)	16 (21%)	0.008
No	28 (100%)	59 (79%)	
**Percutaneous or surgical biopsy prior to LT**			
Yes	0 (0%)	35 (49%)	0.001
No	28 (100%)	36 (51%)	
**Adjuvant therapy**			
Yes	0 (0%)	12 (17%)	0.02
No	28 (100%)	60 (83%)	
**pT classification**			
pT1	1 (4%)	6 (8%)	0.51
pT2	13 (48%)	30 (40%)	
pT3	13 (48%)	35 (47%)	
pT4	0 (0%)	4 (5%)	
**pN classification**			
pN0	28 (100%)	34 (46%)	0.001
pN1	0 (0%)	37 (50%)	
pN2	0 (0%)	3 (4%)	
**Distal bile duct margin tumor free**			
Yes	26 (93%)	66 (89%)	0.58
No	2 (7%)	8 (11%)	
**PSC**			
Yes	6 (24%)	NA	-
No	19 (76%)	NA	
Median time on waiting list in days*	30 (range: 1–870)	NA	-
Median preoperative CA19.9 value in kU/L**	48 (range: 4–1410)	NA	-
**90 Day mortality**			
Yes	3 (11%)	13 (17%)	0.44
No	25 (89%)	64 (83%)	

Abbreviations: LT: liver transplantation, PSC: primary sclerosing cholangitis, NA: not available

*Data available for 25 patients.

**Data available for 16 patients.

### Survival

For survival analyses, patients who had undergone (an attempt at) resection of the tumor prior to transplantation were excluded (n = 34 and missing data: n = 8) because we assumed that in many of those cases liver transplantation was performed because of postoperative liver failure. Actuarial 5-year survival for the entire group of 105 patients that underwent liver transplantation for hilar cholangiocarcinoma was 32%. The 90-day mortality rate was 15%.

**Fig 2:**
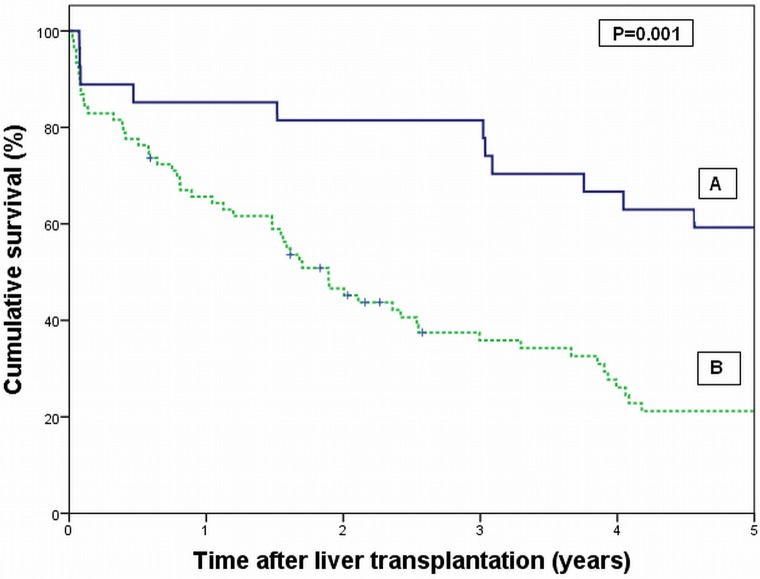
Survival analysis of patients undergoing liver transplantation for hilar cholangiocarcinoma according to patients who complied with the Mayo Clinic criteria for liver transplantation, but were not treated with neo-adjuvant chemoradiotherapy (group A), versus patients who not complied with the Mayo Clinic criteria (group B). P = 0.001 (Log rank test).

Patients who complied with the Mayo Clinic criteria (group A) showed a significant better survival compared to patients not complying with the Mayo selection criteria (group B). The 5-year survival rate was 59% in group A versus 21% in group B (P = 0.001) (Fig 2). In both groups one patient was lost to follow-up, explaining the numbers at risk: 27 patients in group A and 76 in group B. After correction for 90-day mortality (3 patients in group A) a 5-year survival rate of 67% was reached in group A.

We did not perform a comparative analysis between patients from group A and patients that were treated with neo-adjuvant therapy because the neo-adjuvant therapy regimen in this study was not uniform.

### Recurrence of disease

**Fig 3:**
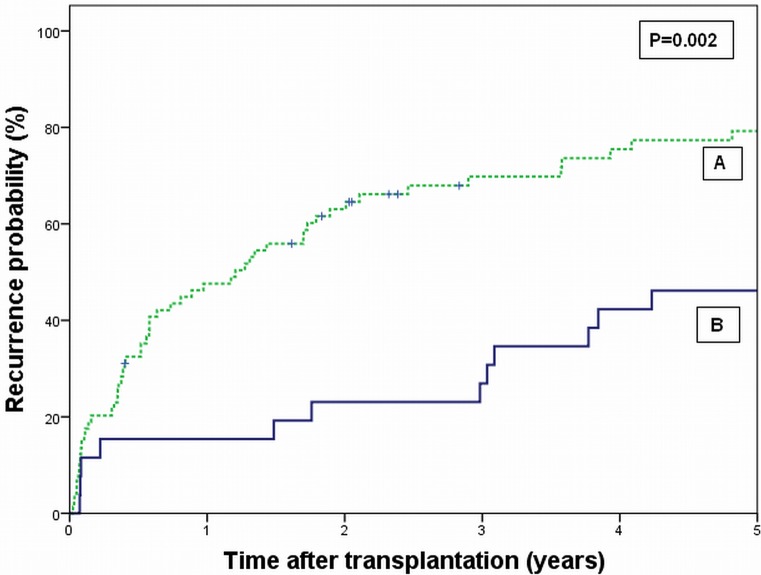
Cumulative probability of recurrence of disease after liver transplantation for hilar cholangiocarcinoma according to patients who complied with the Mayo Clinic criteria for liver transplantation, but were not treated with neo-adjuvant chemoradiotherapy (group A), versus patients who not complied with the Mayo Clinic criteria (group B). P = 0.002 (Log rank test).

Data on recurrence of disease were available for 26 patients in group A and 74 patients in group B. Mean time-to-recurrence was 7.7 (± 6.4) years in group A versus 2.8 (±4.6) years in group B (P = 0.002). Nine of 17 deaths (53%) in group A were not attributable to tumor recurrence whereas in group B 22 of 61 deaths (36%) were unrelated to recurrence. Fig 3 presents the estimated cumulative probability of recurrence over time for the two groups. At 5 years follow-up, the estimated probability of recurrence was significantly lower in group A (46%) compared to group B (79%) (P = 0.002).

### Univariate and multivariate analyses

Results of univariate analysis of variables associated with survival are presented in Table 3. Patients who had undergone (an attempt at) resection of the tumor prior to transplantation were excluded (n = 34 and missing data: n = 8). Only lymph node status was found to be of significant influence on 5-year survival rate (43% in patients with pN0 disease versus 16% in patients with pN1 disease, P = 0.002). The adjusted hazard ratio for positive lymph node status, calculated in a Cox regression analysis, was 2.09 (95%-Confidence interval: 1.31–3.34. P = 0.002).

**Table 3:** Univariate analysis for five-year survival in patients transplanted for hilar cholangiocarcinoma. Thirty four patients who underwent an attempt to surgically remove the tumor prior to transplantation and 8 patients with missing variables were excluded.

Variable	Total number of patients	5-year survival (%)	P-value
**Age (yr)**			
<60	85 (83%)	33	0.24
>60	18 (17%)	25	
**Gender**			
Male	71 (69%)	28	0.35
Female	32 (31%)	42	
**Neo-adjuvant therapy**			
Yes	16 (16%)	34	0.37
Male	85 (84%)	31	
**Adjuvant therapy**			
Yes	12 (12%)	33	0,47
No	86 (88%)	34	
**pT-classisfication**			
Early stage (pT1,2)	50 (50%)	31	0.72
Late stage (pT3,4)	51 (50%)	35	
pN-classification*			
pN0	61 (63%)	43	0.002
pN1	36 (37%)	16	
**Radical resection**			
Yes	90 (90%)	34	0,79
No	10 (10%)	23	

*Three patients with pN2 disease were excluded from the analysis

## Discussion

With the introduction of a neo-adjuvant chemoradiation protocol, liver transplantation for patients with unresectable hCCA or hCCA arising in the setting of PSC has been re-introduced. The concept was pioneered by the team at the University of Nebraska[18] and embraced and modified by the Mayo Clinic group in Rochester. Today more centers are adopting the protocol. [19] However, from the beginning, the question has emerged in the literature whether the strict selection criteria that are being applied to enter the protocol are more important for the success of the program than the neo-adjuvant chemoradiotherapy itself.[13] Further, It should be noted that the use of the protocol is not without risk and is associated with a 40% rate of vascular complications secondary to the high-dose external beam radiation therapy and brachytherapy.[20] This study was undertaken to evaluate the impact of selection alone, without the use of neo-adjuvant therapy, on the outcome of patients transplanted for hilar cholangiocarcinoma in Europe. In this retrospective cohort a five year survival rate of 59% was found in patients that complied with the Mayo Clinic criteria for liver transplantation, but had not undergone neo-adjuvant chemoradiotherapy. To adequately interpret these results, a number of issues should be addressed.

First, not all selection criteria could be directly translated to our cohort. The Mayo Clinic does not accept patients with a tumor size > 3 cm for liver transplantation. Since we had no information about tumor size and tumor size does not correspond to a particular T-stage, we accepted all T-stages in our selected subgroup. In accordance with the Mayo Clinic, we excluded patients with regional lymph node metastases and those who had undergone invasive attempts for tissue diagnosis (surgical or percutaneous biopsy). Endoscopic brushings to confirm the diagnosis was not considered an exclusion criterion. A positive distal bile duct margin was, comparable to the Mayo series, also not an exclusion criterion.

Second, it was shown that the improvement in survival of patients complying with the Mayo Clinic selection criteria (group A) was attributable to a superior oncological outcome, because these patients also demonstrated a significant lower tumor recurrence probability.

Third, the five-year survival rate of 59% in group A is still slightly less than the five-year survival rates of 65–70% reported in the Mayo Clinic series. However, not all patients (around 50%) enrolled in Mayo Clinic series had pathological confirmation of hCCA at the start of neo-adjuvant therapy.[15] It is well known that the diagnosis of hCCA can be difficult because endoscopic brushings or biopsies are often negative or inconclusive.[21] Therefore, the Mayo Clinic group also accepts patients with a malignant-appearing stricture on percutaneous or endoscopic cholangiography and at least one of the three following criteria: polysomy on fluorescent in situ hybridization; or CA-19.9 > 100 U/mL; or a mass on cross-sectional imaging at the site of the stricture. A recent publication from the Mayo Clinic group addressing this issue, showed that patients with pretreatment pathological confirmation of hCCA arising in the setting of PSC, have a significant worse 5-year survival compared to patients without pathological confirmation (66 vs 92%).[15] In patients with hCCA arising de novo, 5-year survival rates in the group with and without pretreatment pathological confirmation were comparable: 63% and 65% respectively. In addition, a recent publication from Duignan et al[22] in which their experience with the Mayo protocol in patients with pathological confirmed hCCA was reported, showed a 4-year survival rate of 60%.These survival rates are similar to the 5-year survival rate of 59% in our subgroup of patients complying with the Mayo criteria.

In our cohort lymph node status was found to be the only significant factor for survival in univariate analysis. We believe that identification of lymph node metastases is probably the most important step in selecting patients with hCCA for liver transplantation.

Obviously, the current study has a number limitations related to its retrospective and multicenter design. First, a response rate of 37% of the contacted centers is not very high, but the 21 centers that did participate, reported 69% of the total amount of patients that were transplanted between 1990 and 2010 according to the ELTR. Because we approached all centers for additional information, we believe the obtained data is very reliable. To achieve the highest possible response rate, we deliberately developed a short questionnaire lowering the threshold for centers to reply. Twenty-seven centers transplanted only one or two patients and because we assumed that the tumors in this group would comprise mainly of incidentalomas, these centers were not vigorously approached in case they did not reply.

Second, 21% of patients in group B underwent neo-adjuvant therapy and 17% underwent adjuvant therapy. Unfortunately it was not possible to conduct analyses between these subgroups and group A, because the (neo-)adjuvant protocols in group B were too heterogeneous. Based on our study, no statements can be made about the effect of (neo-)adjuvant therapy on the outcome of liver transplantation for hCCA.

Third, the present study is based on data from the ELTR. Centers performing liver transplantation report their cases and diagnoses to the ELTR. However, if this has been omitted for any reason, the patient was subsequently lost to our survey.

## Conclusions

In conclusion, this study reports the ELTR experience of liver transplantation for hilar cholangiocarcinoma without the use of neo-adjuvant therapy. It was shown that selection is vital to improve the outcome of these patients. Regional lymph node status was identified as an independent prognostic factor for survival. A subgroup analysis of selected patients, meeting the Mayo Clinic criteria for liver transplantation resulted in a 5-year survival rate of 59% which closely approaches the survival rates of 63–66% reported by the Mayo Clinic for patients with pretreatment pathological confirmation of hCCA. Although the data should be cautiously interpreted because of the retrospective study design, our study suggests that selection is more important than neo-adjuvant therapy. However, the final answer should come from a randomized trial, as was already suggested by Bismuth in 2000.[13]

## Supporting Information

S1 Fig Questionnaire to the participating ELTR centers. (PDF)

S2 Fig Study approval by the ELITA board. (PDF)

S3 Fig Study approval by the Medical Ethics Review Board of the University Medical Center Groningen. (PDF)
