# Supplementary material for: Strict Selection Alone of Patients Undergoing Liver Transplantation for Hilar Cholangiocarcinoma Is Associated with Improved Survival
Source: PLoS One. 2016 Jun 8;11(6):e0156127. doi: 10.1371/journal.pone.0156127 (PMC4898828; doi:10.1371/journal.pone.0156127)
Supplement: S1 Fig — (PDF) [file pone.0156127.s001.pdf]

1. Hilar CCA Survey

PLEASE FILL IN ONE QUESTIONNAIRE FOR EACH PATIENT THAT UNDERWENT LIVER TRANSPLANTATION FOR HILAR CHOLANGIOCARCINOMA.

*1. What is your center's name and what are the patient initials and/or date of birth?

Name of Medical Center

City

Country

Patient initials

Male/Female

Date of birth (please in the format; DD.MM.YYYY)

2. Date of transplantation?

	DD		MM		YYYY
Date		/		/	

*3. What was the indication for liver transplantation?

- Hilar cholangiocarcinoma (CCA)
- Other disease (for example PSC) but hilar cholangiocarcinoma was found incidentally
- Acute liver failure
- If the diagnosis was NOT hilar CCA, you can end the survey for this patient and press "done".

4. Was surgical or percutaneous biopsy of the tumor performed BEFORE transplantation?

- Yes
- No
- Unknown

5. Was there a previous attempt for surgical resection of the tumor BEFORE transplantation?

- Yes
- No
- Unknown

6. Was the patient treated with neo-adjuvant therapy?

- No
- Unknown
- Yes (please specify the type of neo-adjuvant therapy)

7. Was the patient treated with adjuvant therapy?

- ◯ No
- ◯ Unknown
- ◯ Yes (please specify the type adjuvant therapy)

8. What was the definitive histopathological diagnosis? T status:

- ◯ 1
- ◯ 2
- ◯ 3
- ◯ 4

9. What was the definitive histopathological diagnosis? N status:

- ◯ 0
- ◯ 1
- ◯ 2

10. Was the distal bile duct resection-margin tumor free?

- ◯ Yes
- ◯ No

11. Was mTor inhibitor part of immunosuppression in maintenance?

- ◯ No
- ◯ Yes
- ◯ Unknown

12. Has there been evidence for recurrence of hilar CCA after liver transplantation?

- ◯ No
- ◯ Yes, please specify the date of recurrence of hilar CCA (format DD.MM.YYYY)

13. What was the last date of follow-up?

DD MM YYYY

Please fill in the last date of follow-up ___ / ___ / ___

14. What was the status at last follow-up?

- ◯ Alive
- ◯ Dead, please specify date of death (format DD.MM.YYYY))

Thank you for completing this survey!

Participating centers are welcomed as co-author according to the regulations for ELTR-based studies and publications.
