# Supplementary material for: Strict Selection Alone of Patients Undergoing Liver Transplantation for Hilar Cholangiocarcinoma Is Associated with Improved Survival
Source: PLoS One. 2016 Jun 8;11(6):e0156127. doi: 10.1371/journal.pone.0156127 (PMC4898828; doi:10.1371/journal.pone.0156127)
Supplement: S2 Fig — (PDF) [file pone.0156127.s002.pdf]

European Liver and Intestine Transplant Association
A Section of the European Society for Organ Transplantation

and the European Liver Transplant Registry (ELTR)

Chairman
Andrew K. Burroughs

Vice Chairman
Bernard de Hemptinne

Secretary
Paolo Muiesan

Treasurer
Michele Colledan

ELTR Custodian
Rene Adam

Members
William Bennet
Gabriela A. Berlakovich
Christophe Duvoux
Martin Oliverius
Jacques Pirenne
Johann Pratschke

Prof. Robert Porte
Head of Hepato-Pancreato-Biliary Surgery and Liver Transplantation
University Medical Center Groningen
P.O. Box 30.001
9700 RB Groningen
The Netherlands

Subject: ELTR Study Proposal

Monday 20th June, 2011

Dear Robert,

With reference to your e-mail dated 2nd January 2011 proposing a study based on ELTR database on the outcome after liver transplantation for cholangiocarcinoma (CCA) we are very pleased to send official confirmation that we accept this interesting project and that you may therefore make use of the ELTR data.

Best Regards,

Paolo Muiesan	Andrew K. Burroughs	René Adam
ELITA Secretary	ELITA Chairman	ELTR Custodian

Correspondence ELITA
c/o Mr P Muiesan, Consultant HPB and Transplant Surgeon
The Liver Unit, Queen Elizabeth Hospital, Edgbaston, Birmingham, B15 2TH, UK
•Telephone +44 121 627 2418 • Telefax +44 121 414 1833 • E-mail secretary@elita.org • Website http://www.elita.org •
