# Supplementary material for: Strict Selection Alone of Patients Undergoing Liver Transplantation for Hilar Cholangiocarcinoma Is Associated with Improved Survival
Source: PLoS One. 2016 Jun 8;11(6):e0156127. doi: 10.1371/journal.pone.0156127 (PMC4898828; doi:10.1371/journal.pone.0156127)
Supplement: S3 Fig — (PDF) [file pone.0156127.s003.pdf]

P.O. Box 30 001, 9700 RB Groningen, The Netherlands

University Medical Center Groningen

Medical Ethics Review Board

Phone +31(0)50 361 42 04
Fax +31 (0)50 361 43 51
E-mail metc@umcg.nl

Ref. M16.191012

To
Henk-Jan Mantel, MD
Hepatopancreaticobiliaire Chirurgie en
Levertransplantatie
Huispostcode: BA 33

Date: 11th April 2016
Subject: **Strict Selection Alone of Patients Undergoing Liver Transplantation for Hilar Cholangiocarcinoma is Associated with Improved Survival**

Dear Sir,

I, the undersigned, declare that the submission entitled *"Strict Selection Alone of Patients Undergoing Liver Transplantation for Hilar Cholangiocarcinoma is Associated with Improved Survival"* by Mantel et all, fulfils all the requirements for patient anonymity and is in agreement with regulations of our University Hospital for publication of patient data.

Sincerely,

P/o

W.A. Kamps MD PhD
chairman

umcg
